# The Effects of an Eight-Week Swimming Program on Body Composition and Assessment of Dietary Intake in Post-COVID-19 Patients

**DOI:** 10.1155/2024/3037784

**Published:** 2024-03-27

**Authors:** Bostjan Jakše, Barbara Gilić, Marko Đurović, Dorica Šajber

**Affiliations:** ^1^Boštjan Jakše s.p, Radomlje, Slovenia; ^2^University of Split, Faculty of Kinesiology, Split, Croatia; ^3^Faculty of Sport and Physical Education, University of Niš, Niš, Serbia; ^4^Faculty of Sport, University of Ljubljana, Ljubljana, Slovenia; ^5^Swimming Association of Slovenia, Ljubljana, Slovenia

## Abstract

COVID-19 infection and its consequences (long-term COVID-19 syndrome) have implications for weight loss, body composition, and diet quality. In the context of the “PostCovSwim” project, which is part of a broader international study, the impact of an eight-week swimming program on post-COVID-19 patients' nutritional status (i.e., body composition and dietary intake) was evaluated. Body composition and dietary intake were assessed by medically approved and calibrated bioelectrical impedance (Tanita 780 S MA) and food frequency questionnaires. At the baseline, most participants were classified as overweight based on their body mass index (BMI). However, their body fat percentage (BF%) classification indicated normal weight, although females were near obesity thresholds. Furthermore, at the baseline, according to the BMI classification, 62% of females and 61% of males were female, whereas according to the BF% for obesity classification, 44% of females and 43% of males were considered overweight or obese. Surprisingly, despite the eight-week program, there were no significant changes in body composition. Additionally, the assessment of dietary intake, which remained consistent throughout the study, revealed dietary imbalances characterized by an unhealthy low-carbohydrate, high-fat dietary pattern. This dietary pattern entailed excessive consumption of ultraprocessed foods; reduced carbohydrate intake (39% E vs. 37% E); increased total fat intake (46% E vs. 47% E); increased saturated fatty acids (14% E vs. 13% E); increased cholesterol (412 mg/d vs. 425 mg/d); increased free sugars (7% E vs. 7% E); and inadequate intake of fibre (24 g/d vs. 20 g/d), polyunsaturated fatty acids (6.6% E vs. 7.7% E), vitamin B12 (in females: 3.1 *µ*g/d), vitamin C (86 mg/d vs. 66 mg/d), vitamin D (2 *µ*g/d vs. 3.2 *µ*g/d), folate (in males: 258 *µ*g/d), calcium (777 mg/d vs. 743 mg/d), and selenium (in males: 66 *µ*g/d). After an eight-week swimming program following COVID-19, no significant changes were observed in the subjects' body composition. Their dietary intake was found to not align with the dietary recommendations. These findings underscore the urgency of implementing comprehensive dietary and lifestyle interventions for post-COVID-19 patients to optimize their recovery and overall well-being. Physical activity, like a swimming program, may positively affect various aspects of human well-being.

## 1. Introduction

COVID-19, which results from the SARS-CoV-2 virus, has swiftly spread into a global pandemic, impacting millions of people worldwide [1]. While extensive documentation has addressed the acute respiratory symptoms and fatalities linked to COVID-19, an increasing body of evidence has drawn attention to enduring symptoms that extend beyond the acute phase. This phenomenon is commonly termed “long COVID syndrome” or “postacute sequelae of SARS-CoV-2 infection” [2, 3]. Long COVID-19 encompasses a diverse spectrum of symptoms, including persistent fatigue, breathlessness, cognitive deficits, and musculoskeletal discomfort, persisting for weeks, months, and even years following the initial resolution of the infection [4, 5]. The persistence of these symptoms has generated substantial concern regarding the lasting health implications of COVID-19, prompting ongoing research endeavors aimed at comprehending the underlying mechanisms and formulating effective therapeutic strategies [5, 6].

Body mass index (BMI) and body composition have emerged as crucial factors influencing the outcome of COVID-19 and the persistence of long-term COVID-19 symptoms [7]. Numerous studies have established a connection between higher BMI and an increased risk of severe COVID-19, including hospitalization and mortality [6, 8]. Furthermore, individuals with excess body fat, particularly visceral fat and low muscle mass, may be at a greater risk for long COVID-19 symptoms, which can persist for an extended duration postinfection and slower recovery (i.e., to return to typical health and functioning) [9–12]. These findings underscore the importance of considering BMI and body composition when evaluating COVID-19 risk factors and long-term health consequences.

A disease such as COVID-19 can cause inflammation and oxidative stress in the body. In this regard, it is essential to have a healthy diet that includes foods rich in antioxidants and anti-inflammatory compounds. These nutrients can help support the immune system and minimize the adverse effects of inflammation and oxidative stress on overall health [13–15]. Therefore, diet and healthy lifestyles play pivotal roles in both the prevention and management of COVID-19 and its long-term consequences [16–19]. Nutrient-rich, plant-based diets that include plentiful fruits and vegetables, whole grains, legumes, lean proteins, and essential vitamins and minerals can bolster the immune system's resilience against viral infections, potentially reducing the risk of severe COVID-19 [16, 18–20]. Moreover, adopting a balanced and nutritious diet may contribute to the recovery and mitigation of persistent symptoms in long-term COVID patients [3, 16, 20]. Dietary choices can impact inflammation, oxidative stress, and overall health, making them crucial considerations in the comprehensive approach to combating the effects of COVID-19 [17, 21].

Furthermore, physical activity plays a significant role in the management and resolution of long-term COVID-19 symptoms. Engaging in structured exercise programs tailored to individuals' capabilities can help alleviate persistent fatigue, musculoskeletal discomfort, and cognitive impairments commonly associated with long COVID-19 [21]. Regular physical activity contributes to improved cardiovascular health, enhanced lung function, and improved overall well-being, which are crucial aspects of long-term recovery from COVID-19-related symptoms. Evidence suggests that a gradual, guided approach to exercise can be an integral component of rehabilitation for individuals experiencing lingering effects from the virus [22, 23]. A recently published longitudinal study of master swimmers over four years, including during the COVID-19 pandemic, revealed that the body composition of master swimmers who swam before the COVID-19 pandemic did not change due to infection with the virus [24]. However, the open question is whether a structured swimming regimen affects the change (improvement) in body composition in post-COVID-19 patients who were previously included in a systematic swimming program. The potential rationale stems from the premise that increased energy consumption resulting from the structured swimming routine, which represents the intervention study tool involving aerobic and strength exercises, may change body composition.

This study aimed to assess the dietary intake status and changes in body composition of post-COVID-19 patients who participated in a two-month structured swimming program conducted in a warm indoor pool. The most significant improvements in health and well-being are known to occur in people who were previously physically inactive, which is what happens in post-COVID-19 patients. Of note, the primary objective of this international research was to investigate if a two-month specialized water-based exercise program can improve the overall well-being of individuals who have recovered from COVID-19. This includes analyzing the subjects' anthropometric status, functional respiratory capacity, motor skills, overall health, anxiety and stress levels, dietary habits, and sleep quality. Although the study was not designed to look for potentially favourable health benefits of an organized swimming program, we hypothesized favourable changes would be visible, primarily on body mass and body composition.

## 2. Materials and Methods

### 2.1. Study Design

The interventional post-COVID-19 Swim study, known as the “PostCovSwim” project, was a multinational endeavour involving three countries: Slovenia (Ljubljana, Velenje, Kranj), Serbia (Beograd, Niš and Novi Sad), and Croatia (Zagreb and Split). All participants were tested by the same team of experts who travelled to the place of their residence and used the same tests. Questionnaires (e.g., demographic and food frequency questionnaires) were adapted for language differences between each country (note that the Croatian and Serbian languages have some minor differences). The study was a collaborative effort between the Slovenian Swimming Association, Hospital Golnik, Faculty of Sport and Physical Education from Serbia, and a Swimming club from Croatia, with cofunded provided by the European Union [25]. Ethical approval for the research was granted by the Ethical Board of the Faculty of Kinesiology, University of Split, on 6 February 2023 (approval document no. 2181-205-02-05-23-004). The research encompassed initial testing, a training program, and final testing. All participants provided informed consent to be part of the study, and they did not receive any financial compensation for their participation.

### 2.2. Subject

The study included adults who had recovered from COVID-19 and voluntarily signed a declaration expressing their willingness to participate. We enrolled all eligible individuals willing to commit to a two-month exercise program. The program required direct measurements of several variables before and after the intervention program, which took three hours each, and a lecture on completing the questionnaires. The participants were asked to complete the questionnaires at home. The swimming program was freely available and provided for participants included in the study who had recovered from COVID-19 (exclusion criteria). Patients who experienced an acute injury or illness preventing them from participating in the tests were excluded from the research. For calculating sample size, programme G*∗*Power v.3.1. was used for difference between two dependent means (matched pairs), with Alpha probability error of 0.05 and power of 0.95. The calculated sample size was 45. At the beginning of the study, 64 participants were enrolled. However, out of 64, 17 did not finish all initial testing procedures. The initial test in the present study included 47 post-COVID-19 patients (59 ± 14 years of age), 9 of whom had hypertension and three of whom had type 2 diabetes. The final testing points included 31 individuals, 22 females (average age 62 ± 13 years), and 9 males (average age 52 ± 14 years). Among them, 18 were adults (average age 49.4 ± 12.4 years), and 13 were older adults (average age 73.2 ± 5.2 years). Additionally, within the entire sample, data from individuals with disabilities who were otherwise part of the study were excluded from the analysis. Furthermore, 16 participants did not finish the intervention programme and did not participate in the final testing point. Therefore, in total, 31 participants were tested at both initial and final testing points and were actively involved in the intervention. The participants were selected and recruited in each country on a voluntary basis. Precisely, swimming federations, swimming clubs, Faculty of sport, and physical education in each country made several commercials and flyers with the most important information regarding the project and swimming program plan. The main criteria based on which participants were responding to the call were undergoing the COVID-19 illness and having long-COVID conditions (explained in the Introduction section). Thus, inclusion criteria were having long COVID symptoms and being more than 18 years of age. Researchers selected participants who were in line with the inclusion criteria.

### 2.3. Intervention Program

The post-COVID-19 swim programme included an eight-week swimming and water exercise programme free of charge. These 16 sessions, which lasted an hour per unit and were conducted in an indoor pool, took place twice weekly, and participants could engage in them as part of the program. The participants were invited to participate in the study in Slovenia. They were invited through the Swimming Federation of Slovenia, based on the recommendation of Golnik Hospital, and using the third university swimming club, whereas, in Croatia (Zagreb), they were invited through public invitations and the swimming club from Zagreb. In Zagreb (Croatia) and Velenje (Slovenia), the participants participated in the program in deep water, while in Ljubljana and Kranj (Slovenia), the program was conducted in both shallow and deep water. The programme included warm-up exercises, aerobic exercises, mobility training, strength, and flexibility exercises, some of which were conducted in shallow water [26] In essence, the program was divided into two parts, with the dividing line being based on swimming skills and abilities, which are also adjusted for age in post-COVID-19 conditions. However, the swimming program started with a warm-up in the water, which included swimming to warm up and observing technique, followed by various jumps in shallow water, interval swimming, and a focus on swimming technique and exhalation. Ultimately, there were low-intensity swimming and flexibility complex exercises in the water for stability. For further details about the training program format, please refer to the following link: https://www.canva.com/design/DAFtfJ7tLkE/znmzfhG0Wiism_lavpGfOw/edit.

### 2.4. Outcome

#### 2.4.1. Body Composition Measurements

Body height (BH) was assessed by a skilled professional (i.e., sports scientist and/or physician) using a standardized medical approved professional personal floor scale with a stand (Kern, MPE 250K100HM, Kern & Sohn, Balingen, Germany). To determine body composition (excluding the BH), a medically approved and calibrated electrical bioimpedance monitor (Tanita 780 S MA, Tokyo, Japan) was used by a certified and experienced researcher (all measurements were conducted by the same coauthor). Due to the multiple locations of the initial and final measurements in two neighboring countries, we opted for a portable body composition analyser (S) version. The chosen method was the only viable option for carrying out the necessary assessments.

Notably, the results obtained using an 8-electrode body composition analyser in the scientific literature were comparable to those obtained via dual-energy X-ray absorptiometry (DXA), providing accurate total body fat measurements for healthy young adult females and males, regardless of their habitual physical activity level [27, 28], and for adults with severe obesity [29]. However, a validation study of our multifrequency BIA model in generally healthy adults (normal weight, overweight, and obese class 1 and 2) aged between 30 and 65 years is still lacking. Nevertheless, the current method of BIA is cost-effective, user-friendly, and portable and does not expose subjects to radiation [30], making it suitable for large-scale studies within the same ethnic group and across various countries. The obtained body composition data included BH, body mass (BM), body mass index (BMI), total body fat percentage (BF %), fat-free mass (FFM), and phase angle (PhA). BMI was calculated as weight in kilograms divided by the square of height in meters. Before the bioimpedance test, participants were instructed to fast for at least 1 hour, avoid exercise for at least 24 hours, and refrain from urination for at least 30 minutes. Female participants were not measured within three days before or after their menstrual cycle (self-reported).

Furthermore, we assessed BMI status based on BMI categories (normal BMI class: 18.5–24.9 kg/m^2^, overweight BMI class: 25–29.9 kg/m^2^, and obesity BMI class: >30 kg/m^2^) and the BF% classification, both of which were established by the World Health Organization (WHO), where an increased BF% obese class was defined as a BF > 25% in males and >35% in females [31, 32].

#### 2.4.2. Dietary Intake

For dietary intake assessment, a standardized food frequency questionnaire (FFQ) was utilized [33]. The FFQ has been validated for estimating food consumption using 7-day diet records [34]. The FFQ was professionally translated from the original Dutch language to Slovenia and Croatia and has been previously employed in several studies conducted in Slovenia on various populations [35–37]. Food items and ingredients obtained from the FFQ were meticulously entered into the National Open Platform for Clinical Nutrition (OPEN) [38, 39], a web-based application developed by the Jožef Stefan Institute in Slovenia and endorsed by the European Federation of the Association of Dietitians [38]. It is noteworthy that the majority of participants did not use any dietary supplements; hence, these participants were not included in the final analysis.

This FFQ comprises nine frequency categories, ranging from “never” to “more than three times a day.” The FFQ was implemented by transforming the consumption frequency values into daily intake frequencies for all items. The midpoint of each frequency category was utilized to determine the most likely consumption. The food intake data from the FFQ were calculated by multiplying the daily frequency of specific foods by the standard portion size for each food, as recommended by the National Institute of Public Health of Slovenia [40], and by the nutrient amount present in one gram of the food. The daily dietary intake was calculated by adding the nutrient content of each food item. Importantly, the FFQ includes minimally processed, processed, or ultraprocessed products containing sodium (e.g., mayonnaise, butter, lard, ketchup, confectionery, canned beans, cheese, fries, commercial bread, and pastries) [41]. Daily dietary intake was determined by summing the nutrient content of each food item. Food intake data (from the FFQ) were utilized to evaluate energy and nutrient intake, as well as the frequency of food group consumption. We have double-checked the manual method to avoid any possible errors. Finally, for this study, and in consideration of the limitations of the method, the analysis will be restricted by the participants' energy and macronutrient intake and (limited number of) micronutrient intake.

For a comprehensive assessment of the subjects' diet quality, we evaluated their energy and nutrient intake in comparison to the Central European (German (D), Austrian (A), and Swiss (CH)) reference values [42]. Additionally, we assessed the consumption of free sugars, defined by the UK Scientific Advisory Committee on Nutrition (SACN) as less than 5% of daily energy intake [43].

Notably, the present study involved a sensitive patient population, and many measurements were taken, requiring considerable time, energy, and concentration [26]. As a result, it was necessary to perform the initial and final measurements at the exact location based on the training process. Due to these multiple factors, the FFQ method was deemed the most practical choice. However, the subjects were provided with thorough instructions for completing the FFQ. However, detailed instructions were given to the participants to complete the FFQ accurately.

## 3. Statistical Analysis

The Kolmogorov‒Smirnov test was used to examine the normality of the variables, and homoscedasticity was checked with Levene's test. As all variables met the normality of the distribution, means and standard deviations were presented.

Two-way analysis of variance (ANOVA) for repeated measurements was used to determine the effects of the applied intervention, with time (pre- to postintervention) × sex (male, female) and time (pre- to postintervention) × age group (adults, seniors) as the main ANOVA effects and “Time × Group” as the interaction effect for anthropometric and body composition variables. To evaluate the effect sizes of the differences (ESs), partial eta squared (*µ*2) was calculated and interpreted as small (ES: >0.02), medium (ES: >0.13), or large (ES: >0.26). The ES was used to further evaluate the practical significance of the results. Additionally, to evaluate univariate differences between measurements, a *t*-test was used for the dependent samples. To evaluate the sex and age differences in dietary intake, a *t*-test was used for independent samples. Multiple statistical tests were used to calculate in detail and present all relevant data regarding the study's objectives.

The statistical program Statistica 13.5 (TIBCO Software, Inc., Palo Alto, CA, USA) was used for analysis, and a significance level of *p* < 0.05 was used for all calculations.

## 4. Results

### 4.1. Participant Characteristics

The initial test in the present study included 47 post-COVID-19 patients, with an average age of 59 ± 14 years. During the study, 16 individuals dropped out of the study, and the final testing points included 31 individuals, 22 females (average age 62 ± 13 years), and 9 males (average age 52 ± 14 years). Among them, 18 were adults (average age 49.4 ± 12.4 years), and 13 were older adults (average age 73.2 ± 5.2 years). The average number of executed training sessions during the 8-week intervention period was similar among the groups, with no statistically significant differences (13-14 sessions vs. 16 preplanned sessions).

### 4.2. Body Composition Status

Table 1 displays the initial (pretest) and final (posttest) body composition values for the entire study sample, as well as for the female and male, adult and older adult subgroups. Based on the BMI classification, it was found that, on average, participants were classified as overweight in both the pretest and posttest, except for older adults who were classified as obese in the posttest. Regarding BF%, on average, the female and male subgroups were within the normal weight subgroup according to the pretest and posttest. However, regarding the baseline BMI classification, 69% of the total sample was overweight (38%) or obese (31%). More adults fell into this category compared to older adults (77% vs 62%), and a similar distribution was observed between females and males (62% vs 61%). Furthermore, based on the obesity classification using BF%, the proportion of females classified as overweight or obese was 44%, while for males, it was 43%. Overall, after the 8-week swimming program, no significant changes in body composition were observed among the participants, regardless of sex and age stratification.

The results of the two-way analysis for repeated measurements for age and sex groups are presented in Tables 2 and 3. The main effects of “group” were observed for body height (medium ES), body fat (medium ES), fat-free mass (medium ES), total body water (medium ES), and PhA (medium ES). No significant “time” or “interaction” effects were recorded for the age group. Concerning grouping according to sex, significant “group” effects were noted for all variables, while no significant “time” or “interaction” effects were noted.

### 4.3. Dietary Intake

Table 4 provides an overview of the subjects' energy and nutrient intake. Notably, we did not modify the subjects' diets, and they maintained their existing eating patterns throughout the study. In comparison to males, females exhibited a lower average energy intake and consumed less protein (14% of energy vs. 15% of energy). On average, both females and males derived a larger portion of their protein from animal sources, with males consuming 28% more animal protein than plant protein and females consuming 18% more plant protein than animal protein. Total fat intake was comparable between the sexes, exceeding the recommendations for both sexes (46% of energy vs 47% of energy). Carbohydrate intake was 37% of the energy for both sexes. The fibre consumption was below the recommended nutrient intake for both sexes (>33 g/day and >26 g/day for females and males, respectively), with females having a slightly greater intake than men (24 g/day vs 20 g/day). Furthermore, the average intake of saturated fatty acids (SFAs) in both females and males significantly exceeded typical dietary recommendations (up to 7% of total energy intake was recommended), with females accounting for 14% of total energy intake and males accounting for 13%. A similar trend was observed for dietary cholesterol, with females and males consuming 412 mg/day and 425 mg/day, respectively, surpassing the recommended reference intake of less than 300 mg/day. Additionally, both sexes exceeded the recommended limit for free sugar intake, with both females and males consuming 7% of total energy intake, while the reference is less than 5% of total energy intake. Furthermore, the assessment of nutritional intake revealed inadequate consumption of polyunsaturated fatty acids (PUFAs), fibre, vitamin B12 (in females), vitamin C, vitamin D, folate (in males), calcium, and selenium (in males). These findings underscore the importance of dietary improvements to meet recommended nutrient intake.

## 5. Discussion

### 5.1. Main Findings

In our international 8-week intervention study in which post-COVID-19 participants were prescribed to attend the swimming program twice a week (16 sessions in total), we observed that 13-14 sessions were completed. However, we did not detect significant alterations in body composition during this period. Furthermore, our participants had an above-average BMI, indicating that they were overweight on average. However, concerning BF%, the male and female subgroups were classified as having average weight at the pretest and posttest. It is worth noting that the female subgroup was closer to the obesity cut-off.

Furthermore, at the baseline, 69% of the participants were overweight (38%) or obese (31%). Based on the BF% for obesity classification, 44% of females and 43% of males were considered obese. Therefore, it is crucial to recognize that obesity is characterized by the accumulation of surplus body fat, extending beyond excess BMI, an important consideration for individuals falling within the normal BMI range (e.g., those with sarcopenic obesity [44, 45]). Sarcopenic obesity is currently a pressing global public health issue, the prevalence of which is steadily increasing (e.g., affecting up to 42% of adults) [46, 47]. Therefore, it is advisable to employ both classifications in conjunction [48], particularly in smaller intervention studies and cohorts, to comprehensively evaluate individuals' health status.

It is important to note that this aspect of our findings is just one component of a broader study, and the positive impacts of the swimming program can be extrapolated to other aspects of post-COVID patients' functional well-being and overall quality of life. Additionally, our assessment of nutritional intake, which remained unchanged during the study, revealed that the patients were not adhering to a balanced diet that would adequately meet their nutritional needs. More precisely, based on the assessment of nutritional intake and completed FFQs, it can be concluded that the subjects consumed an unhealthy form of a low-carbohydrate, high-fat (LCHF) diet characterized by an excessive consumption of ultraprocessed foods and lower carbohydrate and fibre intake, accompanied by higher total fat, SFA, cholesterol, and free sugar intake.

### 5.2. Body Mass Index, Body Fat Mass, and COVID-19 Incidence Status

Despite our post-COVID-19 participation in regular weekly exercise as part of the intervention program, they did not experience significant weight loss or improvements in body composition over 8 weeks. While altering body mass or body composition is not the primary goal of a swimming intervention program, it could be considered a desirable secondary outcome. Nonetheless, maintaining a normal BMI is of paramount importance not only for overall health but also in the context of COVID-19 and long COVID-19 recovery. A literature review demonstrated that both obesity and the presence of intraabdominal adipose tissue depots are linked to a heightened risk of severe COVID-19, including hospitalization, pulmonary embolism, admission to the intensive care unit, and mortality [12, 49, 50]. In a recent study, a swimming training regimen of three sessions per week, each lasting one hour and applied over 12 weeks, did not lead to significant changes in body composition parameters in females [51]. Significant changes in body mass and body composition, especially when aiming to preserve muscle mass, typically require a combination of aerobic activities and strength training [52], ideally coupled with dietary change [53, 54]. However, as our participants did not receive any dietary recommendations aimed at improving their nutritional intake, this might be the cause of not recording changes in body composition. This latter notion will be further explained in the following paragraphs.

### 5.3. Swimming Programme and COVID-19

Despite the regular physical activity in our study, which is often recommended for maintaining a healthy body weight and composition, significant changes in weight or body composition were not observed over the 8 weeks. It is also noteworthy that the participants were enrolled in the intervention program at the end of winter and spring, when weather conditions improved, and when people tended to spend more time outdoors and engaging in increased physical activity. Consequently, it can be inferred that overall physical activity levels during the intervention program were greater than those at the initial measurements. Nonetheless, our results are in line with the results obtained by other researchers, where a 12-week swimming intervention (3 times a week for 60 minutes) also did not significantly affect body composition parameters [51]. We acknowledge that a systematic review of randomized controlled trials examining the impact of water-based exercise on body composition in adults and older adults yielded significant effects. However, importantly, the results also indicated that training for less than 120 minutes per week and/or with inadequate intensity was insufficient to improve body composition [55]. These findings apply to participants who had contracted COVID-19 and those who had not. Furthermore, a recent study revealed that middle-aged master swimmers did not experience any changes in body composition parameters even during a 4-year period involving multiple COVID-19 lockdowns [24]. Importantly, COVID-19 patients require a modified implementation program in many aspects. Nonetheless, the overall view of the study results is consistent with previous studies involving different demographic factors, including adults, which have consistently demonstrated that the combination of suitable physical activity and a balanced diet yields the most favorable outcomes, particularly when integrated into a healthy and active lifestyle [53, 54, 56–58].

Notably, regular, adequate, and suitable physical activity plays a crucial role in enhancing control over energy and nutrient intake [59]. This is particularly significant in our contemporary lifestyle, where individuals tend to cycle between periods of excess calorie consumption during holidays and then gradually lose only a portion of the accumulated excess body weight [60]. Moreover, regular physical activity provides numerous health benefits, including a reduced risk of more than 25 common chronic noncommunicable diseases and premature death, independent of changes in body composition [61, 62]. The greatest health benefits of physical activity are achieved through a combination of aerobic exercises such as walking, cycling, and swimming, along with strength training, which can include bodyweight exercises, the use of exercise equipment, or gym workouts [63]. Ultimately, the key is to encourage individuals to adopt a healthy and active lifestyle, increasing the likelihood of their participation in organized and guided exercise programs, along with a support system [62].

### 5.4. Dietary Intake and COVID-19

The lack of significant changes in body weight and body composition observed in our study is more likely attributable to an unbalanced diet rather than insufficient exercise frequency or intensity. A healthy BMI plays a crucial role in enhancing the prospects of recovery prospects from the acute phase of COVID-19, potentially reducing the chances of developing long-term COVID-19 with persistent symptoms [12, 49, 50]. This highlights the importance of weight management and adopting a well-balanced diet as integral components of a comprehensive strategy to minimize the effects of COVID-19 and promote a faster recovery. During the COVID-19 pandemic, the adoption of a more plant-based diet has been demonstrated to be a crucial component of a healthy and active lifestyle for preventing adverse health outcomes associated with COVID-19. This includes reducing the incidence of long-term COVID-19 syndrome incidence, the need for hospital treatment, premature mortality, and potential consequences of future coronavirus pandemics [16, 17, 64]. Specifically, a plant-based diet may offer benefits to individuals with a potentially weaker immune response to vaccination, highlighting its supportive role in enhancing the effectiveness of COVID-19 vaccination [64]. A study conducted in six countries a year ago investigated the relationship between dietary patterns and COVID-19 incidence, disease severity, and duration among healthcare workers exposed to COVID-19 patients. These findings indicated that a plant-based diet, including various vegetarian dietary patterns, was associated with a reduced risk of severe COVID-19 infection [16]. Building upon prepandemic research demonstrating the favorable effects of a plant-based diet on various preventive aspects of common chronic noncommunicable diseases, it is suggested that a plant-based diet may offer broad benefits for managing clinical conditions often observed in individuals with COVID-19 [17]. A robust immune system is crucial for protecting the body against harmful pathogens. Malnutrition can weaken the immune system, and the immune system's response to an infection can also affect nutritional status. A lack of certain nutrients can lead to immune system disorders. The consumption of enough vitamins (A, D, B12, C, and E), minerals (selenium, zinc, and iron), and omega-3 fatty acids is crucial for maintaining a healthy immune system [65]. Energy restriction, without nutritional deficiency, is likely key to weight loss and changing body composition [66, 67]. To achieve long-term benefits, a sustainable approach that allows for ad libitum eating while maintaining a moderate energy deficit and sufficient protein intake should be followed [54, 68–70].

For the general population, the review paper on dietary recommendations for post-COVID-19 syndrome patients recommends maintaining adequate energy intake from unprocessed food groups. These findings emphasize the importance of achieving nutritional sufficiency to support the preservation of FFM and aiding in the recovery from low-grade inflammation and associated functional impairments such as fatigue, muscle weakness, loss of appetite, and altered taste perception. The proposed dietary approach leans more toward plant-based foods, unprocessed and lean animal proteins, and plant sources of fats [21].

Importantly, while we did not modify the subjects' diet, attributing changes in body mass and body composition solely to the swimming program, we recognize that, in general, incorporating dietary changes alongside physical activity, such as our swimming program, is advisable for post-COVID-19 patients. Taken together, even though the results of this study did not reveal improvement in body composition, such training interventions are advisable for individuals to reach good health status. Therefore, this study should serve as a guideline for emphasizing the importance of incorporating swimming and water exercise interventions among post-COVID-19 patients and patients recovering from similar lung diseases. Research findings suggest that combining aerobic activity, such as swimming, with “classical” strength training is necessary to improve body composition. The proposed swimming intervention involved strength training, but water-based exercises will not help improve muscle strength, at least not based on our current understanding of this specific motor skill. This should be tailored to the individual, focusing on improving their overall eating habits, including moderate caloric restriction and greater protein intake, without causing nutrient malnutrition.

Although swimming workouts are not meant to be performed independently, they can be used as part of a post-COVID-19 rehabilitation method, as movements in water allow easier physical activity maintenance and appropriate rehabilitation for recovery. Facilitating conditions for physical activity, such as access to water, organized physical activity, and a sense of inclusion in the post-COVID-19 process, positively impacts individuals' motivation levels and the feeling that society cares for the dignity of people during such a global epidemic. At the same time, we understand that patients often succumb to fate and passively wait for time to heal the consequences of an illness. Therefore, we believe that it is crucial to provide people from different socioeconomic backgrounds with accessible or even free exercise programs, even if they do not always result in optimal improvements in all areas pursued.

Of importance, this study is part of a larger research project and shows only a part of the effects that swimming intervention can produce. Future manuscripts from the same research group could evaluate all other aspects of health indicators, including muscle strength, endurance, and lung capacity, in post-COVID-19 patients. The conclusions and recommendations will be possible when the results of the entire comprehensive study are processed and published.

## 6. Strengths and Limitations

The study's strengths lie in its international intervention nature, featuring numerous standardized and valid measurements, as well as high patient engagement. However, weaknesses stemmed from the smaller pool of participants involved in the two-month organized swimming training and the even smaller number of participants who completed the study (i.e., participated in posttesting). Thus, the results of this study should be interpreted with caution.

Nevertheless, these data hold significant importance due to the substantial burden post-COVID-19 patients place on health and social systems. This study may be useful in clinical practice because COVID-19 patients may maintain a healthy lifestyle, which includes physical activity (e.g., swimming workouts) and a recommended diet. Furthermore, public health agencies should provide clear recommendations and guidelines, including a healthy diet and physical activity. Consequently, studies and programs of this nature warrant broader community support.

Future studies should show the effects of a swimming exercise programme on several health status indicators, including body composition, cardiovascular health, respiratory health, and muscle performance indicators. In addition, we suggest combining methods not commonly used in intervention science because the changes obtained are difficult to attribute to the proportions of individual interventions used. However, for post-COVID-19 rehabilitation, we recommend testing a multidisciplinary comprehensive support system. This training involved a swimming program, “classical” strength training, breathing exercises/meditation, a change in diet, and psychological support. The state should be interested in implementing this approach, as it would significantly reduce the direct and indirect costs associated with the previous epidemic.

## 7. Conclusion

No significant body mass loss or body composition improvements were observed after an eight-week intervention program for post-COVID-19 patients that involved regular weekly exercise. The BMI and BF% obesity classifications did not yield similar proportions of subjects in each class. Initially, there was a difference between the percentage of females and males classified as overweight or obese based on BMI and BF%. When BMI was used, initially, 62% of females and 61% of males were classified as overweight or obese, while when BF% was used, only 44% of females and 43% of males were classified as overweight or obese. Therefore, for a comprehensive understanding of individuals' nutritional status, particularly in the context of varying COVID-19 severity, it is advisable to employ both BMI and BF% obesity classifications simultaneously.

The evaluation of dietary intake among the study participants revealed that, on average, they maintained an inadequate diet, contributing to an overweight BMI status, with a more pronounced issue among females than males (according to the BF% obesity classification). An unhealthy diet cannot be offset solely by physical activity (i.e., swimming). However, a combination of a balanced diet and appropriate physical activity (the type, combination, frequency, and intensity) can lead to comprehensive impacts on overall health and well-being.

Nevertheless, any level of physical activity, even at this frequency and intensity, is superior to complete inactivity, and it is essential to acknowledge that physical activity yields additional health benefits beyond what is discussed in this study, which will be elaborated upon in forthcoming research.

## Figures and Tables

**Table 1:** Changes in body composition status.

Variables	Total (*n* = 31)		Female (*n* = 22)		Male (*n* = 9)		Adult (*n* = 18)		Older adult (*n* = 13)	
	Pretest	Posttest	Pretest	Posttest	Pretest	Posttest	Pretest	Posttest	Pretest	Posttest
BH (cm)	168.2 ± 12.1	168.2 ± 12.1	162.6 ± 7.8	162.6 ± 7.8	181.4 ± 10.7	181.4 ± 10.7	171.3 ± 13.1	171.3 ± 13.1	163.9 ± 9.6	163.9 ± 9.6
BM (kg)	76.3 ± 15.4	76.2 ± 15.2	72.5 ± 14.8	72.4 ± 14.5	85.5 ± 13.5	85.5 ± 13.2	76.7 ± 16.9	76.6 ± 16.6	75.7 ± 13.6	75.6 ± 13.6
BMI (kg/m^2^)	27.0 ± 4.2	27.1 ± 4.1	27.6 ± 4.7	27.6 ± 4.7	25.9 ± 2.4	25.8 ± 2.3	26.1 ± 3.4	26.2 ± 3.3	28.3 ± 4.9	28.2 ± 4.9
BF (%)	30.4 ± 8.7	30.6 ± 8.5	34.5 ± 6.8	34.6 ± 6.4	21.05 ± 4.0	21.2 ± 3.9	27.4 ± 8.6	27.6 ± 8.2	34.5 ± 7.3	34.5 ± 7.4
FFM (kg)	53.1 ± 11.3	53.1 ± 11.5	47.2 ± 5.7	47.1 ± 5.7	66.9 ± 8.7	67.1 ± 8.9	56.1 ± 12.5	56.1 ± 12.7	49.2 ± 8.4	49.1 ± 8.5
TBW (%)	48.1 ± 7.9	48.6 ± 6.3	45.7 ± 5.8	45.9 ± 5.2	53.5 ± 6.6	54.9 ± 3.4	50.3 ± 6.7	50.9 ± 5.6	45.2 ± 6.4	45.6 ± 5.9
PhA (°)	5.7 ± 0.7	5.7 ± 0.6	5.4 ± 0.6	5.4 ± 0.5	6.2 ± 0.6	6.2 ± 0.4	5.9 ± 0.6	5.9 ± 0.5	5.3 ± 0.5	5.3 ± 0.5

BH: body height; BM: body mass; BMI: body mass index; BF: body fat; FFM: fat-free mass; TBW: total body water; PhA: phase angle.

**Table 2:** Results of the two-way analysis for repeated measurements for age groups (adults and older adults).

Variables	Main effects						Interaction		
	Group			Time			(Group × time)		
	*F* test	*p*	*µ*2	*F* test	*p*	*µ*2	*F* test	*p*	*µ*2
BH (cm)	5.29	0.03	0.17	—	—	—	—	—	—
BM (kg)	0.51	0.48	0.02	0.34	0.57	0.02	0.3	0.59	0.01
BMI (kg/m^2^)	1.85	0.19	0.07	—	—	—	—	—	—
BF (%)	7.05	0.01	0.22	0.23	0.64	0.01	0.03	0.86	0.01
FFM (kg)	5.14	0.03	0.17	0.03	0.87	0.01	0.01	0.97	0.001
TBW (%)	6.98	0.01	0.22	0.54	0.47	0.02	0.08	0.78	0.01
PhA (°)	9.87	0.01	0.28	0.44	0.52	0.02	0.44	0.52	0.02

BH: body height; BM: body mass; BMI: body mass index; BF: body fat; FFM: fat-free mass; TBW: total body water; PhA: phase angle. *µ*2—partial eta squared values–effect size.

**Table 3:** Results of the two-way analysis for repeated measurements for sex groups (female, male).

Variables	Main effects						Interaction		
	Group			Time			(Group × time)		
	*F* test	*p*	*µ*2	*F* test	*p*	*µ*2	*F* test	*p*	*µ*2
BH (cm)	29.48	0.001	0.5	—	—	—	—	—	—
BM (kg)	5.36	0.03	0.16	0.11	0.74	0.01	0.08	0.76	0.01
BMI (kg/m^2^)	1.09	0.3	0.04	0.03	0.87	0.001	0.03	0.87	0.001
BF (%)	32.13	0.001	0.53	0.44	0.51	0.02	0.01	0.94	0.001
FFM (kg)	55.44	0.001	0.66	0.05	0.82	0.01	0.54	0.47	0.02
TBW (%)	17.43	0.001	0.38	0.9	0.35	0.03	0.51	0.48	0.02
PhA (°)	14.37	0.001	0.34	0.01	0.92	0.001	0.01	0.92	0.001

BH: body height; BM: body mass; BMI: body mass index; BF: body fat; FFM: fat-free mass; TBW: total body water; PhA: phase angle. *µ*2—partial eta squared values–effect size.

**Table 4:** Energy and nutrient intake.

Variable (/d)	Female	Male	*p* value
E (kcal)	2049 ± 443	2204 ± 387	0.30
Protein plant (g)	26 ± 10	22 ± 7	0.27
Protein animal (g)	47 ± 20	60 ± 3	0.09
SFA (g)	32 ± 10	33 ± 8	0.82
MUFA (g)	35 ± 15	36 ± 10	0.91
PUFA (g)	15 ± 10	19 ± 8	0.30
Total fat (g)	105 ± 30	115 ± 24	0.32
Cholesterol (mg)	412 ± 279	425 ± 230	0.88
Free sugar (g)	36 ± 24	39 ± 23	0.65
Total sugar (g)	89 ± 40	85 ± 32	0.75
Carbohydrates (g)	198 ± 54	205 ± 60	0.70
Fibre (g)	24 ± 6	20 ± 6	0.06
Vitamin A (mg)	4.4 ± 2.6	4.0 ± 2.5	0.66
Vitamin B12 (*µ*g)	3.1 ± 1.8	4.1 ± 2.3	0.13
Vitamin C (mg)	86 ± 44	66 ± 40	0.19
Vitamin D (*µ*g)	2.0 ± 1.4	3.2 ± 2.5	0.06
Vitamin E (mg)	16 ± 7	18 ± 5.8	0.53
Vitamin K (mg)	169 ± 102	127 ± 103	0.23
Folate (*µ*g/d)	304 ± 124	258 ± 102	0.26
Potassium (mg)	2954 ± 966	2683 ± 642	0.38
Ca (mg)	777 ± 296	743 ± 304	0.74
Fe (mg)	14 ± 4	16 ± 10	0.33
Zn (mg)	11 ± 4	11 ± 3	0.93
Se (*µ*g)	65 ± 33	66 ± 24	0.90
Water (ml)	1835 ± 328	1549 ± 271	0.01

E: energy intake; SFA: saturated fatty acids; MUFA: monounsaturated fatty acids; PUFA: polyunsaturated fatty acids.

## Data Availability

The data used to support the findings of this study are included within the article.
